# 快速滤过型净化法结合气相色谱-串联质谱法快速筛查代用茶中125种农药残留

**DOI:** 10.3724/SP.J.1123.2024.09009

**Published:** 2025-07-08

**Authors:** Quan ZHANG, Yutian WU, Lei PENG, Shan BI, Yibing ZHOU, Ye LIN, Liya LIU, Qingyuan CHEN, Xue ZHOU

**Affiliations:** 1.贵州省疾病预防控制中心，贵州 贵阳 550004; 1. Guizhou Center for Disease Control and Prevention，Guiyang 550004，China; 2.贵州医科大学药学院，贵州 贵阳 550025; 2. School of Pharmacy，Guizhou Medical University，Guiyang 550025，China

**Keywords:** 气相色谱-串联质谱, 快速滤过性净化, 农药残留, 快速筛查, 代用茶, gas chromatography-tandem mass spectrometry （GC-MS/MS）, multi-plug filtration cleanup, pesticide residues, rapid screening, tea substitutes

## Abstract

利用快速滤过型净化法结合气相色谱-串联质谱（GC-MS/MS）分析，建立了代用茶基质中125种不同极性农药残留的快速筛查方法。目标物经DB-1701MS石英毛细管色谱柱（30 m×0.25 mm×0.25 μm）程序升温分离，GC-MS/MS多反应监测（MRM）模式检测，基质匹配内标法定量。通过GC-MS/MS检测方法对代用茶中的125种代表性农药进行了方法学验证。结果表明，M-PFC TD-1型茶叶快速滤过型净化柱对铁皮石斛花提取液净化效果良好，而且能保证较高的农药回收率。方法学考察结果表明：125种组分在0.01～1.0 mg/L范围内呈良好的线性关系（*R* ^2^>0.980），方法的检出限（LOD，*S/N*=3）为0.003~0.02 mg/kg，方法的定量限（LOQ，*S/N*=10）为0.01~0.05 mg/kg，在低、中、高3个不同加标水平下125种目标物的回收率范围为62.6%~107.6%，相对标准偏差（RSD）为1.0%~13.8%（*n*=6）。与其他经典的前处理方法相比，本方法无需活化、平衡、洗脱步骤，消耗溶剂较少，且M-PFC TD-1柱在净化效果方面表现得更好，可直接吸附代用茶基质中的色素、生物碱等大分子干扰物，操作简单、快速，灵敏度较高，适用于大批量代用茶中农药多残留的快速筛查。采用本研究建立的方法对贵州省食药物质试点生产企业提供的50份代用茶样品进行检测，其中在2份代用茶样品中各检出至少1种农药残留，检出率为4%。本研究为掌握代用茶产品中的农药残留种类及水平，提高相关的地方标准指标设置或技术要求的科学性和实用性提供了技术支持。

## Abstract (en)

A rapid screening method for 125 pesticide residues of different polarities in tea substitutes matrix was developed using rapid screening combined with gas chromatography-tandem mass spectrometry （GC-MS/MS）. The targets were separated on DB-1701MS quartz capillary column （30 m×0.25 mm×0.25 μm） with programmed temperature rise， detected by GC-MS/MS in multiple reaction monitoring （MRM） mode， and quantified by matrix-matched solution internal standard method. The methodological validation of 125 representative pesticides in tea substitutes was carried out by GC-MS/MS method. The results showed that the tea samples could be extracted most efficiently when using acetonitrile without immersion in water. The M-PFC TD-1 column had a good purification effect on the *Dendrobium officinale* flower extract with a guaranteed high recovery. The results of optimized analysis showed that the 125 components had a good linear correlation in the range of 0.01 mg/L to 1.0 mg/L （*R* ^2^>0.980）. The LODs and LOQs were 0.003-0.02 mg/kg （*S/N*=3） and 0.01-0.05 mg/kg （*S/N*=10），respectively. The recoveries of the 125 targets at three levels were in the range of 62.6%-107.6% with relative standard deviations （RSDs， *n=*6*）* of 1.0%-13.8%. Compared with other classical pretreatment methods， this method does not require activation， equilibration and elution steps， consumes less solvent， and the M-PFC TD-1 column shows better purification effect， which can directly adsorb large molecular interferences， such as pigments and alkaloids， in the matrix of the substitute tea， and is easy to operate， rapid， and with high sensitivity， which is suitable for the rapid screening of pesticide multicomponent residues in the bulk substitute tea. The method established in this study was used to monitor 50 samples of tea substitutes provided by the pilot production enterprises in Guizhou Province， in which at least one pesticide residue was detected in two samples of tea substitutes， with a detection rate of 4%. This study aims to enhance the scientific and practical aspects of setting local standard indicators or technical requirements by understanding the types and levels of pesticide residues in substitute tea products.

代用茶是指采用除茶树鲜叶之外的食用植物原料，如芽叶、花朵及花蕾、果实、根茎等，经过特定加工制作后，以类似于茶叶冲泡的方式来供人们饮用的产品；依据原料种类的差异，代用茶可分为叶类、花类、果类、根茎类以及混合类等多种类型^［1，2］^。当前，我省代用茶类产品种类繁多，涵盖天麻、灵芝、石斛等，但产品质量差异显著，且缺乏有效的监管机制。尽管存在供销合作行业标准GH/T 1091-2014《代用茶》^［3］^和2018年农业农村部行业标准NY/T 2140-2015《绿色食品 代用茶》^［4］^作为现行有效标准，同时贵州、吉林、广西等地也已制定食品安全地方标准，然而至今尚未出台统一的国家标准来规范代用茶产品，导致叶类、花类、果（实）类等代用茶原料中农药残留超标问题频发，严重影响了代用茶产品的整体质量保障。样品前处理阶段是农药残留分析中的关键环节，其耗时约占整个分析流程的2/3^［5，6］^。尽管当前存在多种基质净化填料，但每种单一填料仅能针对特定成分进行净化，而具备广谱净化能力、能有效处理百余种不同极性农药的混合填料及其优化配比却较为少见。此外，农药筛查工作强调前处理过程的简便性、快速性和易操作性，尤其是在处理大量样品时，采用流水线作业方式尤为重要。同时，必须确保净化效果，以避免因净化不彻底导致的假阳性结果，从而减少重复实验验证的次数^［7］^。快速滤过型净化（multi-plug filtration cleanup，m-PFC）法是基于QuEChERS方法开发的一种新型快速样品前处理净化柱^［8］^，通过在多壁碳纳米管（MWCNTs）表面键合不同的官能团或配合物，改善其溶解性和分散性，增强干扰化合物的去除效果，从而大大缩短净化时间，大幅提高前处理总体效率，目前已用于人参、果蔬中多种农药残留检测^［9-11］^。

在针对本省代用茶产品进行农药残留筛查的过程中，我们发现天麻、灵芝、石斛类产品的基质构成相对复杂，尤其是石斛类产品，即便经过前处理步骤，净化后的溶液颜色依然较深。尽管我们在前期的研究中已经对比了Sin-QuEChERS Nano方法与几种经典的样品前处理方法（包括SPE、dSPE、QuEChERS法）在净化效果上的差异^［12］^，但随着新型快速滤过型净化柱的广泛应用，这些净化柱各自展现出不同的产品优势。然而，哪种快速滤过型净化柱更适合用于处理铁皮石斛花这类基质复杂的代用茶产品，目前尚缺乏文献提供明确的数据支持。基于此，本研究对当前常用的前处理方法进行了比较与优化，确定了M-PFC TD-1柱在代用茶等复杂基质净化中的优势。进而，我们建立了快速滤过柱结合气相色谱-串联质谱（GC-MS/MS）分析的方法，用于快速筛查代用茶中的125种农药残留。这些监测目标涵盖了有机氯类、有机磷类、菊酯类等代表性农药，同时也包括了代用茶原材料种植过程中常用的杀菌剂、杀虫剂等。本研究旨在揭示我省代用茶产品中的农药残留种类及水平，为推动国家相关标准的出台、贯彻与实施提供有力支持，并为今后的标准修订工作提出了科学的参考建议。

## 1 实验部分

### 1.1 仪器、试剂与材料

布鲁克Scion-TQ气相色谱-三重四极杆质谱联用仪（美国Bruker公司）；FW100型高速万能粉碎机（天津市泰斯特仪器有限公司）；GL-22MS型高速冷冻离心机（上海卢湘仪离心机仪器有限公司）。

125种定制混合标准溶液（3支套标，分别含43种、42种、42种组分，1.2 mL，溶剂丙酮，各组分质量浓度均为100 mg/L，上海安谱实验科技股份有限公司）；内标（IS）环氧七氯（10 mg/瓶，纯度＞99.8%，德国Dr. Ehrenstorfer公司）。乙腈、丙酮、正己烷（色谱纯，纯度均>99.8%，美国TEDIA公司）。M-PFC TD-1型茶叶快速滤过型净化柱（300 mg，2.5 mL，含150 mg MgSO_4_、50 mg PSA、50 mg FeN-MWCNTs磁性氨基化中空纳米材料和50 mg C_18_-3EC，北京科德诺斯技术有限公司）。

代用茶样品由2022年贵州省食药物质试点生产企业提供，其中包含铁皮石斛花、苦荞茶、金银花茶、苦丁茶、灵芝茶等。企业所在地分别隶属贵阳市、遵义市、毕节市、黔东南州、铜仁市、黔西南州、黔南州共7个行政区域。

### 1.2 混合标准溶液配制

分别准确移取3支标准溶液各1 mL混匀，然后用丙酮定容至10 mL，配制成1.0 mg/L混合标准溶液，于‒4 ℃保存，临用前用乙酸乙酯逐级释成质量浓度为0.01~1.0 mg/L的混合标准工作溶液。空白基质溶液在经氮气吹干后，分别加入1 mL上述混合标准溶液进行复溶。随后，再加入40 μL质量浓度为10 mg/L的环氧七氯内标液（溶剂为丙酮），从而配制成一系列基质匹配的混合标准工作溶液。

### 1.3 样品前处理

取250 g代表性样品，利用大功率涡轮粉碎机粉碎，过二号筛（24目）后置于自封袋中备用。

准确称取1.00 g代用茶试样于50 mL离心管中，加入3 mL水，静置30 min，涡旋振荡3 min，加入10 mL 1%乙酸乙腈，涡旋振荡5 min，加入缓冲盐萃取包，涡旋振荡5 min，10 000 r/min离心5 min，离心后在50 mL离心管内吸取1.2~1.5 mL上清液至M-PFC TD-1柱中，匀速推动推杆将上清液过滤至2 mL离心管中，10 000 r/min离心5 min，取 1 mL上清液加入40 μL质量浓度为10 mg/L的环氧七氯内标液，过0.22 μm滤头后待GC-MS/MS分析。其他前处理方法如Sin-QuEChERS Nano法、MPFC-QuEChERS法、M-PFC C（复杂基质）法及d-SPE法参照文献［^12^］的前处理步骤进行操作。

空白样品的制备：经GC-MS/MS验证无目标物的空白铁皮石斛花基质用于下述实验中。

### 1.4 色谱-质谱分析条件

#### 1.4.1 色谱条件

色谱柱：Agilent DB-1701MS石英毛细管色谱柱（30 m×0.25 mm×0.25 μm）；载气：高纯氦气，纯度≥99.999%；碰撞气（CID）：高纯氩气，纯度≥99.999%；分流模式：不分流进样；进样口温度：250 ℃；流速：1 mL/min，进样量：1.0 μL。程序升温条件：起始温度60 ℃，保持1 min，以40 ℃/min升至120 ℃，再以5 ℃/min升至280 ℃，保持9.5 min，共44.0 min。

#### 1.4.2 质谱条件

电离模式：电子轰击电离（EI）；扫描模式：多反应监测（MRM）；离子源温度和传输线温度分别为200 ℃和250 ℃，溶剂延迟时间为7.0 min。目标物的保留时间及质谱参数见表1。

**表1:** 125种目标物及内标的保留时间及质谱参数

No.	Pesticide	*t*_R_/min	Chemical formula	*M*_r_	CAS No.	Ion pairs （*m/z*）	CEs/eV
IS	heptachlor epoxide （环氧七氯）	23.080	C_10_H_5_Cl_7_O	389.3	1024-57-3	353>263^*^， 353>282	15， 15
1	isoprocarb （异丙威）	7.481+14.510	C_11_H_15_O_2_N	193.2	2631-40-5	121>77^*^， 136>121	20， 10
2	dichlorvos （敌敌畏）	7.986	C_4_H_7_Cl_2_O_4_P	221.0	62-73-7	185>109^*^， 185>63	20， 15
3	fenobucarb （仲丁威）	8.710	C_12_H_17_NO_2_	207.3	3766-81-2	121>77^*^， 121>104	20， 15
4	methamidophos （甲胺磷）	9.925	C₂H₈NO₂PS	141.1	10265-92-6	141>95^*^， 141>79	10， 15
5	dichlorobenzonitrile（敌草腈）	10.208	C_7_H_3_Cl_2_N	172.0	1194-65-6	171>100^*^， 171>136	25， 15
6	molinate （禾草敌）	12.643	C_9_H_17_NOS	187.3	2212-67-1	126>55^*^， 126>83	10， 5
7	tecnazene （四氯硝基苯）	14.387	C_l4_C_6_HNO_2_	260.9	117-18-0	259>201^*^， 259>141	10， 30
8	ethoprophos （灭线磷）	15.308	C_8_H_19_O_2_PS_2_	242.3	13194-48-4	158>97^*^， 158>81	18， 15
9	diphenylamine （二苯胺）	15.393	C_12_H_11_N	169.2	122-39-4	169>168^*^， 169>167	10， 15
10	phorate （甲拌磷）	16.413	C₇HO₂PS_3_	260.4	298-02-2	260>231^*^， 260>171	10， 20
11	chlorpropham （氯苯胺灵）	16.668	C_10_H_12_ClNO_2_	213.7	101-21-3	213>127^*^， 213>171	20， 10
12	sulfotep （治螟磷）	16.700	C_8_H_20_O_5_P_2_S_2_	322.3	3689-24-5	322>266^*^， 322>146	10， 10
13	pentachloronitrobenzene （五氯硝基苯）	17.688	C₆Cl₅NO_2_	295.3	82-68-8	249>214^*^， 237>143	15， 20
14	terbufos （特丁硫磷）	17.874	C_9_H_21_O_2_PS_3_	288.4	13071-79-9	231>129^*^， 231>175	20， 10
15	clomazone （异噁草酮）	18.021	C_12_H_14_ClNO_2_	239.7	81777-89-1	125>89^*^， 204>107	15， 20
16	triallate （野麦畏）	18.066	C_10_H_16_Cl_3_NOS	304.7	2303-17-5	268>184^*^， 142.9>83	20， 15
17	diazinon （二嗪磷）	18.159	C_12_H_21_N_2_O_3_PS	304.3	333-41-5	304>137^*^， 304>164	35， 35
18	fonofos （地虫硫膦）	18.312	C_10_H_15_OPS_2_	246.3	944-22-9	246>109^*^， 246>137	18， 10
19	pyrimethanil （嘧霉胺）	18.355	C_12_H_13_N_3_	199.3	53112-28-0	198>156^*^， 198>182	25， 18
20	simazine （西玛津）	19.129	C₇HClN_5_	201.7	122-34-9	201>138^*^， 201>172	10， 15
21	dicloran （氯硝胺）	19.175	C_6_H_4_Cl_2_N_2_O_2_	207.0	99-30-9	176>148^*^， 206>176	10， 10
22	atrazine （莠去津）	19.227	C_8_H_14_ClN_5_	221.7	1912-24-9	215>200^*^， 215>172	10， 15
23	terbuthylazine （特丁津）	19.313	C_9_H_16_ClN_5_	229.7	5915-41-3	214>104^*^， 214>119	15， 10
24	iprobenfos （异稻瘟净）	19.831	C_13_H_21_O_3_PS	288.3	26087-47-8	204>91^*^， 201>122	10， 15
25	triazophos （三唑磷）	19.976	C_12_H_16_N_3_O_3_PS	313.0	24017-47-8	257>119^*^， 257>134	25， 20
26	pronamide （炔苯酰草胺）	20.030	C_12_H_11_Cl_2_NO	256.1	23950-58-5	254>226^*^， 254>191	10， 15
27	pirimicarb （抗蚜威）	20.108	C_11_H_18_N_4_O_2_	238.3	23103-98-2	166>96^*^， 166>83	10， 15
28	dimethoate （乐果）	20.498	C_5_H_12_NO_3_PS_2_	229.3	60-51-5	125>79^*^， 125>93	10， 40
29	chlorpyrifos-methyl （甲基毒死蜱）	20.499	C_7_H_7_Cl_3_NO_3_PS	322.5	5598-13-0	286>241^*^， 286>208	25， 10
30	monocrotophos （久效磷）	20.665	C_7_H_14_NO_5_P	223.2	6923-22-4	127>109^*^， 127>95	15， 18
31	phosphamidon （磷胺）	20.665+22.432	C_10_H_19_ClNO_5_P	299.7	13171-21-6	264>193^*^， 127>109	8，12
32	acetochlor （乙草胺）	20.896	C_14_H_20_ClNO_2_	269.8	34256-82-1	146>131^*^， 146>130	20， 20
33	alachlor （甲草胺）	21.312+25.131	C_14_H_20_ClNO_2_	269.8	15972-60-8	188>160^*^， 188>130	10， 40
34	Prometryn （扑草净）	21.374	C_10_H_19_N_5_S	241.4	7287-19-6	242>184^*^， 242>199	10， 10
35	pirimiphos-methy （甲基嘧啶磷）	21.470	C_11_H_20_N_3_O_3_PS	305.3	29232-93-7	290>125^*^， 290>151	20， 20
36	ametryn （莠灭净）	21.579	C_9_H_17_N_5_S	227.3	834-12-8	227>58^*^， 227>170	10， 10
37	vinclozolin （乙烯菌核利）	21.735	C_12_H_9_Cl_2_NO_3_	286.1	50471-44-8	212>172^*^， 212>145	15， 20
38	thiobencarb （禾草丹）	21.737	C_12_H_16_ClNOS	257.8	28249-77-6	125>89^*^，125>99	20， 11
39	metribuzin （嗪草酮）	21.826	C_7_H_11_N_3_O_2_	169.2	21087-64-9	198>82^*^， 198>55	18， 30
40	metalaxyl （甲霜灵）	21.934	C_15_H_21_NO_4_	279.3	57837-19-1	206>132^*^， 206>162	20， 10
41	chlorpyrifos （毒死蜱）	22.058	C_9_H_11_Cl_3_NO_3_PS	350.6	2921-88-2	314>258^*^， 314>166	15， 35
42	parathion-methyl （甲基对硫磷）	22.332	C_8_H_10_NO_5_PS	263.2	298-00-0	263>109^*^， 263>246	15， 5
43	dicofol （三氯杀螨醇）	22.564	C_14_H_9_Cl_5_O	370.5	115-32-2	251>139^*^， 251>111	10， 35
44	metolachlor （异丙甲草胺）	22.598	C_15_H_22_ClNO_2_	283.8	51218-45-2	162>133^*^， 238>162	15， 10
45	methoprene （烯虫酯）	22.807	C_19_H_34_O_3_	310.5	40596-69-8	111>55^*^， 153>111	15， 5
46	malathion （马拉硫磷）	22.904	C_10_H_19_O_6_PS_2_	330.4	121-75-5	173>99^*^， 173>117	18， 10
47	fenthion （倍硫磷）	22.918	C_10_H_15_O_3_PS_2_	278.3	55-38-9	278>109^*^， 278>125	20， 18
48	cyprodinil （嘧菌环胺）	23.094	CHN_3_	225.3	121552-61-2	224>208^*^， 224>118	18， 40
49	fenitrothion （杀螟硫磷）	23.230	C_9_H_12_NO_5_PS	277.2	122-14-5	260>125^*^， 260>109	15， 10
50	ethofumesate （乙氧呋草黄）	23.354	C_13_H_18_O_5_S	286.3	26225-79-6	286>207^*^， 286>161	10， 20
51	triadimefon （三唑酮）	23.675	C_14_H_16_ClN_3_O_2_	293.8	43121-43-3	208>181^*^， 208>127	10， 15
52	pendimethalin （二甲戊灵）	23.880	C_13_H_19_N_3_O_4_	281.3	40487-42-1	251>162^*^， 252>118	10， 30
53	parathion （对硫磷）	23.909	CHNO₅PS	291.3	56-38-2	291>109^*^， 291>137	15， 10
54	isofenphos-methyl （甲基异柳磷）	24.003	C_14_H_22_NO_4_PS	331.4	99675-03-3	241>199^*^， 199>121	10， 10
55	quinalphos （喹硫磷）	24.467	C_12_H_15_N_2_O_3_PS	298.3	13593-03-8	298>156^*^， 298>129	10， 30
56	isocarbophos （水胺硫磷）	24.552	C_11_H_16_NO_4_PS	289.3	24353-61-5	230>212^*^， 230>197	5， 10
57	propanil （敌稗）	24.648	C_9_H_9_Cl_2_NO	218.1	709-98-8	161>90^*^， 161>126	20， 15
58	penconazole （戊菌唑）	24.648	C_13_H_15_Cl_2_N_3_	284.2	66246-88-6	248>157^*^， 248>192	25， 15
59	phorate sulfoxide （甲拌磷亚砜）	25.039	C_7_H_17_O_3_PS_3_	276.4	2588-03-6	199>143^*^， 97>65	10， 20
60	phorate sulfone （甲拌磷砜）	25.040	C_7_H_17_O_4_PS_3_	292.4	2588-04-7	125>97^*^， 153>97	5， 10
61	fenothiocarb （苯硫威）	25.063	C_13_H_19_NO_2_S	253.4	62850-32-2	160>72^*^， 160>106	10， 12
62	fosthiazate （噻唑膦）	25.100	C_9_H_18_NO_3_PS_2_	283.3	98886-44-3	195>103^*^， 195>139	10， 5
63	tetraconazole （四氟醚唑）	25.127	C_13_H_11_Cl_2_F_4_N_3_O	372.2	112281-77-3	336>218^*^， 336>204	15， 40
64	butachlor （丁草胺）	25.135	C_17_H_26_ClNO_2_	311.8	23184-66-9	237>160^*^， 237>188	5， 10
65	terbufos sulfone （特丁硫磷砜）	25.696	C_9_H_21_O_4_PS_3_	320.4	56070-16-7	153>97^*^， 199>97	10， 20
66	triadimenol （三唑醇）	25.805	C_14_H_18_ClN_3_O_2_	295.8	55219-65-3	168>70^*^， 128>65	10， 25
67	procymidone （腐霉利）	25.942	C_13_H_11_Cl_2_NO_2_	284.1	32809-16-8	283>96^*^， 283>177	10， 30
68	profenofos （丙溴磷）	26.061	CHBrClO₃PS	373.6	41198-08-7	339>269^*^， 339>251	10， 25
69	methidathion （杀扑磷）	26.101	C_6_H_11_N_2_O_4_PS_3_	302.3	950-37-8	145>85^*^， 145>58	10， 15
70	pretilachlor （丙草胺）	26.150	C_17_H_26_ClNO_2_	311.8	51218-49-6	162>147^*^，176>134	15， 15
71	napropamide （敌草胺）	26.156	C_17_H_21_NO_2_	271.4	15299-99-7	271>72^*^， 271>128	10， 5
72	hexaconazole （己唑醇）	26.244	C_14_H_17_Cl_2_N_3_O	314.2	79983-71-4	214>152^*^， 214>124	25， 25
73	oxadiazon （噁草酮）	26.419	C_15_H_18_Cl_2_N_2_O_3_	345.2	19666-30-9	258>175^*^，258>112	10， 25
74	fluazifop-butyl （吡氟禾草灵）	26.696	C_19_H_20_F_3_NO_4_	383.4	69806-50-4	383>282^*^， 383>254	10， 30
75	imazalil （抑霉唑）	26.798	C_14_H_14_Cl_2_N_2_O	297.2	35554-44-0	215>173^*^， 215>145	5， 20
76	paclobutrazol （多效唑）	26.800	C_15_H_20_ClN_3_O	293.8	76738-62-0	236>125^*^， 236>132	10， 5
77	isoprothiolane （稻瘟灵）	26.956	C_12_H_18_O_4_S_2_	290.4	50512-35-1	290>118^*^， 290>204	15， 10
78	phosfolan （硫环磷）	27.076	C_7_H_14_NO_3_PS_2_	255.3	947-02-4	196>140^*^， 168>140	10， 5
79	bupirimate （乙嘧酚磺酸酯）	27.430	C_13_H_24_O_3_N_4_S	316.5	41483-43-6	273>108^*^， 273>150	18， 10
80	oxyfluorfen （乙氧氟草醚）	27.743	C_15_H_11_CIF_3_NO_4_	361.7	42874-03-3	300>223^*^， 300>167	20， 25
81	flutolanil （氟酰胺）	27.885	C_17_H_16_F_3_NO_2_	323.3	66332-96-5	173>145^*^， 281>173	15， 10
82	ethion （乙硫磷）	28.139	C_9_H_22_O_4_P_2_S_4_	384.5	563-12-2	231>129^*^， 231>175	25， 10
83	quinoxyfen （喹氧灵）	28.255	C_15_H_8_Cl_2_FNO	308.1	124495-18-7	307>237^*^， 307>272	20， 10
84	diniconazole （烯唑醇）	28.755	C_15_H_17_Cl_2_N_3_O	326.2	83657-24-3	267>232^*^， 267>232	10， 10
85	piperonyl butoxide （增效醚）	28.765	C_19_H_30_O_5_	338.4	51-03-6	176>131^*^， 176>117	10， 18
86	trifloxystrobin （肟菌酯）	28.777	C_20_H_19_F_3_N_2_O_4_	408.4	141517-21-7	222>162^*^， 222>190	10， 5
87	cyproconazole （环丙唑醇）	28.845	C_15_H_18_ClN_3_O	291.8	94361-06-5	222>125^*^， 139>111	15， 15
88	myclobutanil （腈菌唑）	28.856	C_15_H_17_ClN_4_	288.8	88671-89-0	179>125^*^， 179>129	15， 20
89	benalaxyl （苯霜灵）	28.875	C_20_H_23_NO_3_	325.4	71626-11-4	206>132^*^， 206>162	18， 10
90	diclofop-methyl （禾草灵）	29.352	C_15_H_12_Cl_2_O_4_	327.2	51338-27-3	253>162^*^， 340>253	15， 10
91	edifenphos （敌瘟磷）	29.416	C_14_H_15_O_2_PS_2_	310.4	17109-49-8	173>109^*^， 310>173	10， 15
92	fipronil （氟虫腈）	29.424	C_12_H_4_Cl_2_F_6_N_4_OS	437.1	120068-37-3	367>213^*^， 367>215	30， 25
93	propiconazole （丙环唑）	29.508	C_15_H_17_Cl_2_N_3_O_2_	342.2	60207-90-1	259>173^*^， 259>145	20， 40
94	epoxiconazole （氟环唑）	29.683+31.022	C_17_H_13_ClFN_3_O	329.8	133855-98-8	192>138^*^， 192>111	10， 25
95	fenthion sulfoxide （倍硫磷亚砜）	29.824	C_10_H_15_O_4_PS_2_	294.3	3761-41-9	278>109^*^， 278>169	15， 15
96	isazofos （氯唑磷）	29.834	C_9_H_17_ClN_3_O_3_PS	313.7	42509-80-8	161>119^*^， 161>103	10， 15
97	fenthion sulfone （倍硫磷砜）	30.462	C_10_H_15_O_5_PS_2_	310.3	3761-42-0	311>105^*^， 311>137	20， 20
98	bromopropylate （溴螨酯）	30.755	C_17_H_16_Br_2_O_3_	428.1	18181-80-1	341>185^*^， 341>183	15， 15
99	etoxazole （乙螨唑）	30.931	CHF₂NO₂	359.4	153233-91-1	359>187^*^， 359>340	15， 12
100	iprodione （异菌脲）	30.931	C_13_H_13_Cl_2_N_3_O_3_	330.2	36734-19-7	314>245^*^， 314>56	10， 25
101	oxadixyl （噁霜灵）	30.995	C_14_H_18_N_2_O_4_	278.3	77732-09-3	163>132^*^， 163>117	5， 25
102	fludioxonil （咯菌腈）	31.003	C_12_H_6_F_2_N_2_O_2_	248.2	131341-86-1	248>182^*^， 248>154	10， 15
103	fenpropathrin （甲氰菊酯）	31.152	C_22_H_23_NO_3_	349.4	39515-41-8	265>210^*^， 265>181	10， 20
104	tebuconazole （戊唑醇）	31.272	C_16_H_22_ClN_3_O	307.8	107534-96-3	250>125^*^， 250>153	15， 10
105	pyriproxyfen （吡丙醚）	31.420	C_20_H_19_NO_3_	321.4	95737-68-1	136>96^*^， 131>78	15， 20
106	anilofos （莎稗磷）	31.420	C_13_H_19_ClNO_3_PS_2_	367.9	64249-01-0	226>157^*^， 226>157	15， 10
107	fenamidone （咪唑菌酮）	32.206	C_17_H_17_N_3_OS	311.4	161326-34-7	238>237^*^， 268>180	10， 20
108	pyridaphenthion （哒嗪硫磷）	32.210	C_14_H_17_N_2_O_4_PS	340.3	119-12-0	340>199^*^， 340>109	10， 20
109	phosmet （亚胺硫磷）	32.436	C_11_H_12_NO_4_PS_2_	317.3	732-11-6	160>77^*^， 160>105	25， 18
110	bifenox （甲羧除草醚）	32.642	C_14_H_9_Cl_2_NO_5_	342.1	42576-02-3	341>189^*^， 341>281	20， 20
111	tetradifon （三氯杀螨砜）	32.655	C_12_H6Cl4O2S	356.1	116-29-0	229>201^*^， 229>16	15， 20
112	lambda-cyhalothrin （高效氯氟氰菊酯）	33.090+33.461	C_23_H_19_ClF_3_NO_3_	449.9	91465-08-6	181>152^*^， 181>127	20， 30
113	phosalone （伏杀硫磷）	33.479	C_12_H_15_ClNO_4_PS_2_	367.8	2310-17-0	367>182^*^， 367>111	10， 30
114	permethrin （氯菊酯）	33.765	C_21_H_20_Cl_2_O_3_	391.3	52645-53-1	183>168^*^， 183>152	10， 20
115	mefenacet （苯噻酰草胺）	33.825	C_16_H_14_N_2_O_2_S	298.4	73250-68-7	192>136^*^， 192>109	15， 30
116	fenarimol （氯苯嘧啶醇）	33.849	C_17_H_12_Cl_2_N_2_O	331.2	60168-88-9	330>139^*^， 330>111	10， 30
117	pyridaben （哒螨灵）	34.271	C_19_H_25_ClN_2_OS	364.9	96489-71-3	309>147^*^， 309>132	12， 25
118	coumaphos （蝇毒磷）	36.824	C_14_H_16_ClO_5_PS	362.8	56-72-4	362>109^*^， 362>226	15， 15
119	cyfluthrin （氟氯氰菊酯）	37.053	CHCl₂FNO_3_	434.3	68359-37-5	206>151^*^， 206>177	15， 20
120	cypermethrin （氯氰菊酯）	37.089	C_22_H_19_Cl_2_NO_3_	416.3	52315-07-8	163>91^*^， 163>127	10， 5
121	flucythrinate （氟氰戊菊酯）	37.930	C_26_H_23_F_2_NO_4_	451.5	70124-77-5	225>147^*^， 225>118	10， 25
122	fenvalerate （氰戊菊酯）	40.018	CHClNO_3_	419.9	51630-58-1	225>119^*^， 225>91	18， 25
123	fluvalinate （氟胺氰菊酯）	41.132	CHClF_3_N_2_O_3_	502.9	102851-06-9	250>55^*^， 250>200	20， 20
124	difenoconazole （苯醚甲环唑）	41.782	C_19_H_17_Cl_2_N_3_O_3_	406.3	119446-68-3	323>265^*^， 323>201	15， 30
125	deltamethrin （溴氰菊酯）	42.334	C_22_H_19_Br_2_NO_3_	505.2	52918-63-5	253>172^*^， 253>199	10， 25

* Quantitative ion pair. CEs： collision energies.

## 2 结果与讨论

### 2.1 称样质量及提取溶剂的选择

代用茶样品通常含水量较低，基质较为复杂，茶叶中农药残留检测一般取样量均小于2 g，基于此，本研究为了保证样品均匀性和目标农药的检测灵敏度，分别比较了铁皮石斛花空白样品不同称样质量（0.50、1.00、1.50、2.00 g）对检测结果的影响。结果表明：当取样量为2.00 g时，加水后样品膨胀体积较大，提取液不能完全浸没，影响提取效率，同时样品净化液颜色较深，基质干扰严重；当取样量为0.50 g时样品均匀性受影响，同时方法的定量限达不到相关要求；当取样量为1.00、1.50 g时对结果的影响差别不大，因此，本方法为了取样操作便捷，同时尽可能减少样品的基质效应，最终称样量确定为1.00 g。

用于农药提取的有机溶剂有很多，但根据目标组分的极性不同，相关研究中通常使用乙腈、丙酮作为提取溶剂^［13］^。本研究选取6个铁皮石斛花空白样品，每种提取溶剂做2个平行样品，分别考察不同提取溶剂（丙酮、乙腈与1%乙酸乙腈）的提取效果。结果表明：当用丙酮作为提取溶剂时样品提取液的颜色较深，样品基质中因过多的杂质（如色素）被提取出来而对后续的净化操作带来一定难度，同时，经丙酮提取后的提取液体积明显变少，影响最终的定量结果，而用乙腈或1%乙酸乙腈的提取效果优于丙酮。

在以上6个铁皮石斛花空白样品中添加0.5 mL的1 mg/L混合标准溶液后放置过夜，其他均按照1.3节操作，分别考察不同提取溶剂（丙酮、乙腈与1%乙酸乙腈）对目标物平均提取回收率的影响，不同溶剂提取铁皮石斛花中125种农药在不同回收率范围的数量分布图见图1。

**图1:**
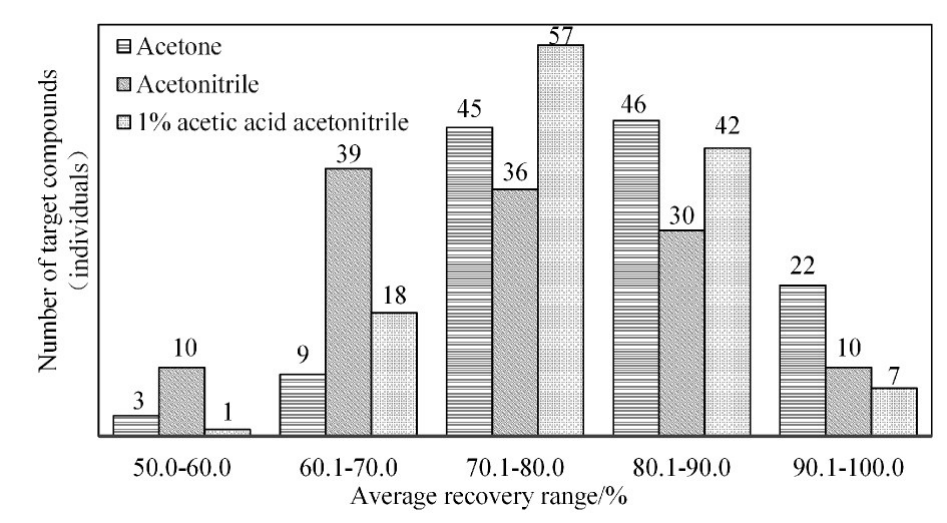
不同溶剂提取铁皮石斛花中125种农药在不同回收率范围的数量分布图

3种提取溶剂（丙酮、乙腈与1%乙酸乙腈）提取125种农药的平均回收率分别为80.8%、74.2%、77.7%，以丙酮和1%乙酸乙腈作为提取溶剂，目标组分在回收率为60.0%~100.0%范围内的数量分别占比97.6%、99.2%，两种溶剂的提取率相差不大，综合上述实验结果，本研究中提取溶剂最终确定为1%乙酸乙腈。

### 2.2 净化效果比较与优化

代用茶中富含茶多酚、脂肪酸、咖啡因和色素等物质，可以和待测目标物共提取，不仅干扰目标物的分离和测定，而且污染仪器，因而简便有效的净化方式至关重要^［14］^。目前常用的快速净化工具主要有Sin-QuEChERS Nano柱、MPFC-QuEChERS柱、M-PFC C柱、M-PFC TD-1柱和d-SPE萃取包及纯化管，其中Sin-QuEChERS Nano柱含900 mg MgSO_4_、45 mg MWCNTs、90 mg PSA和15 mg GCB），MPFC-QuEChERS柱复杂基质超滤柱（3 mL）含150 mg MgSO_4_、25 mg PSA和15 mg GCB）、M-PFC C柱（200 mg，2.5 mL）含150 mg MgSO_4_、30 mg PSA和20 mg FeN-MWCNT磁性氨基化中空纳米材料），d-SPE萃取包内填料为6.0 g MgSO_4_及1.5 g NaOAc，纯化管内含150 mg MgSO_4_、50 mg PSA、50 mg GCB和50 mg C_18_。本研究选取6份铁皮石斛花空白样品，分别根据1.3节进行前处理操作，在全扫描模式下（扫描范围：*m/z* 50~500）分别上机测定，以净化效果、总离子流色谱图分别比较未净化、Sin-QuEChERS Nano柱、MPFC-QuEChERS柱、M-PFC C柱、M-PFC TD-1柱和d-SPE法的净化效果，采用5种净化方式净化后的溶液见图2，总离子流色谱图见图3。

**图2:**
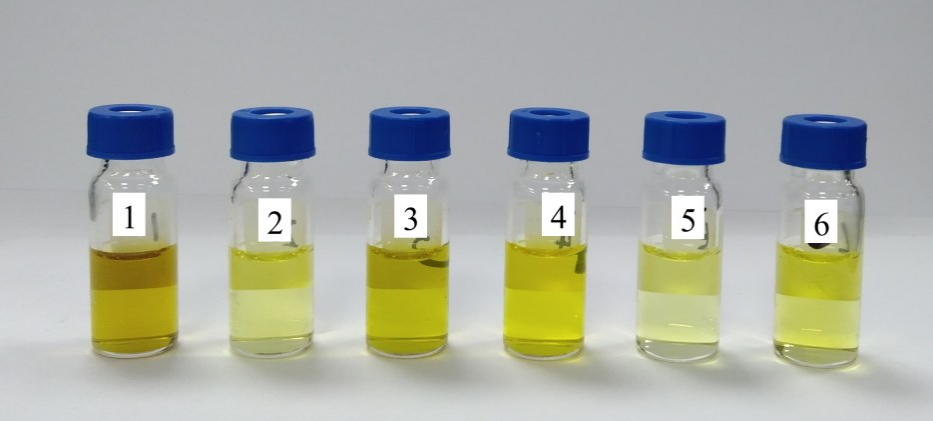
采用5种净化方式净化后的溶液照片

**图3:**
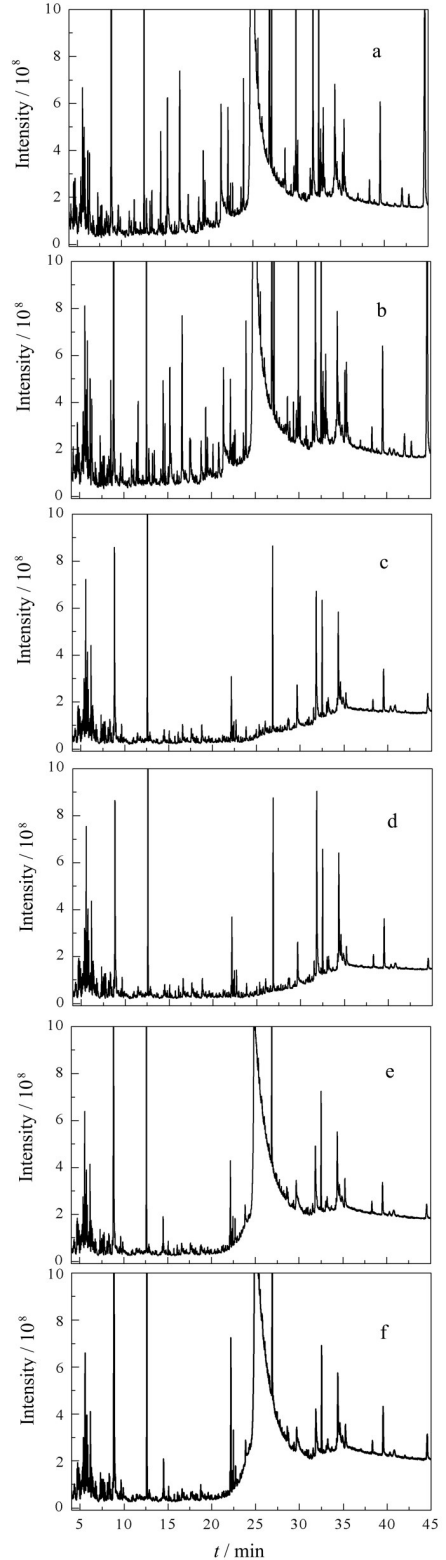
经不同净化方式处理后的铁皮石斛花提取液在全扫描模式下的总离子流色谱图

从图2和图3可以看出，经Sin-QuEChERS Nano柱和M-PFC TD-1柱净化后的溶液颜色较浅，都可有效降低杂质干扰，净化效果较好，其中125种目标物分别在10 μg/kg和100 μg/kg水平下经M-PFC TD-1柱净化后的基质效应（ME）范围在100.0~121.0内的数量最多（见表2），所以本研究最终确定以M-PFC TD-1柱作为代用茶样品的净化柱，铁皮石斛花加标样品的总离子流图见图4。其次，为了验证该净化柱的适用性，本研究选取了不同基质代用茶（灵芝、天麻、石斛等）进行实验考察，结果表明样品的基质净化效果及加标回收率结果均满足相关要求。

**表2:** 5种净化方式下铁皮石斛花中125种农药在两个加标水平下不同基质效应范围内的数量分布

Matrix effect/%	100 μg/kg						10 μg/kg					
	Unpurified	Sin-QuEChERS Nano	MPFC-QuEChERS	M-PFC C	M-PFC TD-1	d-SPE	Unpurified	Sin-QuEChERS Nano	MPFC-QuEChERS	M-PFC C	M-PFC TD-1	d-SPE
0-100.0	8	7	10	7	11	9	5	6	8	11	9	10
100.0-121.0	7	5	15	15	38	24	9	18	17	23	35	25
121.0-151.0	14	54	57	65	56	63	24	50	54	55	53	58
151.0-201.0	80	45	35	29	18	26	74	41	43	34	27	31
201.0-300.0	16	14	8	9	2	3	13	10	3	2	1	1

**图4:**
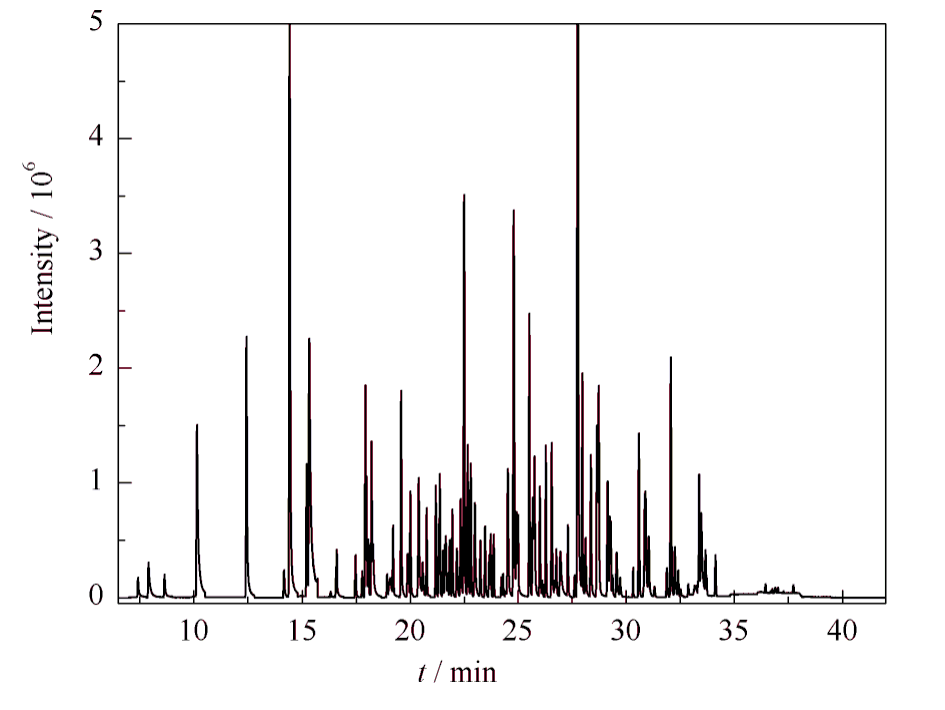
铁皮石斛花加标样品的总离子流图

### 2.3 基质效应

质谱定量检测分析中首要考虑的就是基质效应问题，由于杂质的干扰它会造成增强或抑制目标组分的信号响应，严重影响定量的准确性^［15，16］^。一般采用相对响应值法评价基质效应，ME=基质标准溶液的峰面积/溶剂标准溶液的峰面积×100%。当ME的值为80%~120%时，基质效应可忽略不计，当ME<80%，表现为基质抑制效应，当ME>120%，表现为基质增强效应^［17，18］^。本研究分别取10个批次不同生产厂家的50 g铁皮石斛花空白样品进行混匀，制备成混合铁皮石斛花样品基质，通过空白铁皮石斛花基质加标（10 μg/kg、100 μg/kg）和溶剂加标2种方法，分别按照1.3节中的5种方式进行操作，每种方式平行测定5次，当结果满足RSD<20%时取其均值代入公式计算基质效应，经5种净化方式下铁皮石斛花中125种农药在不同基质效应范围内的数量分布见表2。

从上述结果看出，铁皮石斛花基质效应干扰比较严重，大多数目标物的基质效应均是以增强为主，如未净化时大部分目标物的基质效应为151.0%~201.0%，很难准确地进行目标物定量分析。在空白基质添加溶剂标准，在10 μg/kg和100.0 μg/kg的水平下，5种净化方式中只有M-PFC TD-1柱净化后的基质效应在100.0%~121.0%内的目标物数量较多，净化优势相对明显，这表明M-PFC TD-1柱中FeN-MWCNTs磁性氨基化中空纳米材料可以有效去除杂质，减小基质效应。为了更有效地降低基质效应对定量的影响，后续实验采用基质匹配工作曲线内标法定量。

### 2.4 线性范围、检出限、定量限及加标回收率

取铁皮石斛花空白样品分别加入6个不同浓度的混合标准溶液，在1.4节GC-MS/MS条件下进行测定。以目标物的定量离子峰面积和内标物的定量离子峰面积的比值为纵坐标、目标物标准溶液质量浓度和内标物质量浓度的比值为横坐标，绘制线性曲线。

同时，采用在铁皮石斛花空白样品中添加目标物的方法，以3倍和10倍信噪比（*S/N*）对应的含量确定检出限（LOD）和定量限（LOQ）。

为评价方法的准确度与精密度，取数份铁皮石斛花空白样品，分别添加20、50、500 μL质量浓度为1.0 mg/L的混合标准使用液，放置1 h后平行测定6次。结果表明，125种目标物的线性关系良好（相关系数*R* ^2^>0.98）；采用加标回收的方法确定方法的检出限及定量限分别为0.003~0.02 mg/kg和0.01~0.05 mg/kg；125种化合物的加标回收率为62.6%~107.6%，RSD（*n=*6）为1.0%~13.8%；相关数据见附表1（www.chrom-China.com）。说明本方法重复性良好，可满足实际检测的需要。

### 2.5 实际样品检测

采用本研究建立的方法对贵州省食药物质试点生产企业提供的50份代用茶样品进行检测，其中在2份铁皮石斛花代用茶样品中各检出至少1种农药残留，检出率为4%，分别是戊唑醇（0.25 mg/kg）、甲基对硫磷（0.015 mg/kg）、高效氯氟氰菊酯（0.18 mg/kg）；为验证本方法的准确性，应用国标方法GB 23200.113-2018中7.1.3章节对阳性样品进行复检，结果为戊唑醇0.23 mg/kg、甲基对硫磷0.018 mg/kg、高效氯氟氰菊酯0.21 mg/kg，检测结果无明显差异，表明本方法准确度较高。阳性样品中各目标物的MRM色谱图如图5所示。其中杀菌剂戊唑醇、杀虫剂高效氯氟氰菊酯均属于登记农药，检出量均大于0.1 mg/kg，这可能是在铁皮石斛人工栽培种植及运输过程中为了防止出现炭疽病、白绢病、黑斑病、黑腐病、软腐病和枯萎病等病害，以及蜗牛、蛞蝓、斜纹夜蛾或红蜘蛛虫害等问题时喷洒农药导致残留，与之前文献报道的检测结果相一致^［19］^；按照现有的标准来看，检出农药的残留量均未超过国家标准GB 2763-2021^［20］^及地方标准DBS 52/045-2020规定的最大残留限量值^［21］^，表明铁皮石斛种植过程相对规范，但同时也提示我们在杀虫剂、杀菌剂的使用中应予以限制和指导，规范合理用药，加强代用茶产品质量安全监管。

**图5:**
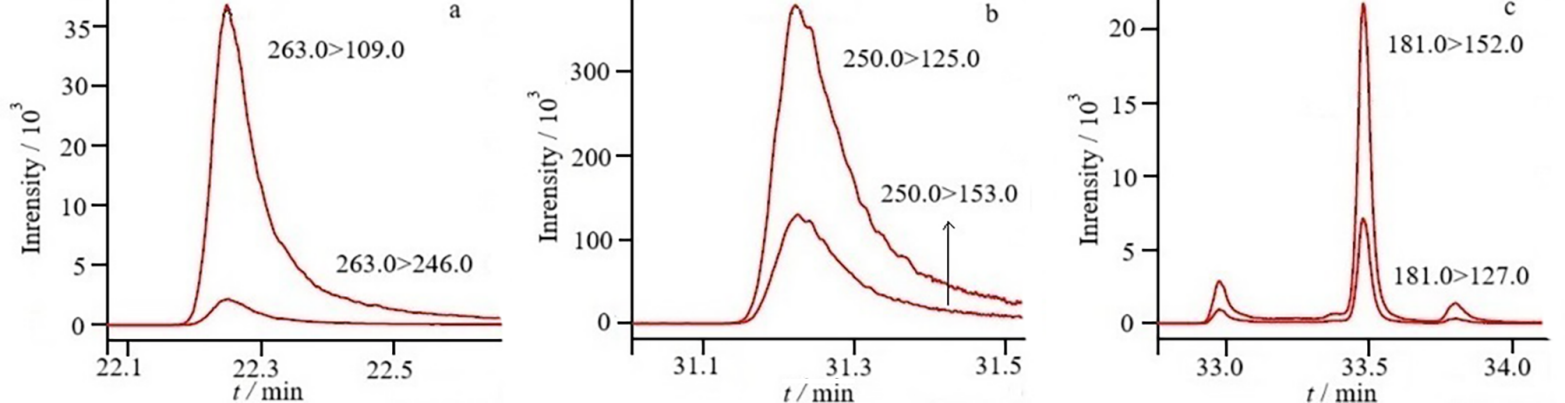
阳性样品中各目标物的MRM色谱图

## 3 结论

本研究建立了M-PFC TD-1型茶叶快速滤过型净化柱结合GC-MS/MS快速筛查铁皮石斛花等复杂基质代用茶中多组分农药残留的方法。该快速滤过型净化柱在净化效果方面表现优于现在主流方法，操作简单快捷，可对代用茶产品中农药残留进行高通量快速筛查，具有实际应用价值。
